# Systems biology approach to identification of biomarkers for metastatic progression in cancer

**DOI:** 10.1186/1471-2105-9-S9-S8

**Published:** 2008-08-12

**Authors:** Andrey A Ptitsyn, Michael M Weil, Douglas H Thamm

**Affiliations:** 1 Center for Bioinformatics, Department of Microbiology, Immunology and Pathology, Colorado State University, Colorado, USA; 2 Department of Environmental and Radiological Health Sciences, Colorado State University, Colorado, USA; 3 Animal Cancer Center, Department of Clinical Sciences, Colorado State University, Colorado, USA

## Abstract

**Background:**

Metastases are responsible for the majority of cancer fatalities. The molecular mechanisms governing metastasis are poorly understood, hindering early diagnosis and treatment. Previous studies of gene expression patterns in metastasis have concentrated on selection of a small number of "signature" biomarkers.

**Results:**

We propose an alternative approach that puts into focus gene interaction networks and molecular pathways rather than separate genes. We have reanalyzed expression data from a large set of primary solid and metastatic tumors originating from different tissues using the latest available tools for normalization, identification of differentially expressed genes and pathway analysis. Our studies indicate that regardless of the tissue of origin, all metastatic tumors share a number of common features related to changes in basic energy metabolism, cell adhesion/cytoskeleton remodeling, antigen presentation and cell cycle regulation. Analysis of multiple independent datasets indicates significantly reduced oxidative phosphorylation in metastases compared to primary solid tumors.

**Conclusion:**

Our methods allow identification of robust, although not necessarily highly expressed biomarkers. A systems approach relying on groups of interacting genes rather than single markers is also essential for understanding the cellular processes leading to metastatic progression. We have identified metabolic pathways associated with metastasis that may serve as novel targets for therapeutic intervention.

## Introduction

Metastasis (originating from Greek *μετισταναι*, to change) is the single most important event changing the course of cancer from manageable to fatal. For metastasis to occur, tumor cells must acquire the ability to detach from the original tumor, relocate through the blood or lymphatic circulation and start a new colony in a different part of the organism [1]. In spite of intensive research [2-11] there is no consensus regarding the origin of metastases. According to one model, metastatic transformation can be triggered in primary solid tumors by certain conditions, while another model links metastatic potential to a very rare subtype of tumor cells, occurring on the order of one in many millions. Genetic background is also viewed as an important determining factor in metastatic transformation [10,11]. This difference is important for both diagnostic and prognostic purposes.

Early cancer is clinically heterogeneous, and many patients can have an "indolent" disease course that does not significantly affect their survival. Once metastatic disease is documented clinically, the majority of patients die from their tumors as opposed to other causes [12]. This has led some researchers to consider the disease as a series of "states" that include clinically localized tumors and those that have metastasized, as a framework to assess the clinical and biological factors associated with specific phenotypes and outcomes [13]. However, there are other plausible concepts. Analysis of relations between different molecular subtypes of cancer and identification of genes specific to such subtypes is important for understanding the biological basis for this clinical heterogeneity and thus is critical in assessing prognosis, selecting therapy, and evaluating treatment effects. Metastatic transformation is a multi-stage process involving complex interactions between tumor cells and the host [14]. Cells from primary tumors must detach, invade stromal tissue, and penetrate blood or lymphatic vessels by which they disseminate. They must survive in the circulation to reach a secondary site in which they lodge because of physical size or binding to specific tissues. To form clinically significant tumors, metastatic cells must also adjust their metabolism and signaling systems to proliferate in the new microenvironment. Tumor cells growing at metastatic sites are continually selected for their growth advantage. This is a complex and dynamic process that is expected to involve alterations in many genes and transcriptional programs.

Considerable amounts of gene expression data have been deposited in public databases and/or are available upon request from other investigators. Analysis of these data is generally limited to one set at a time. However, recent years have seen multiple attempts to conduct meta-analysis across independent data sets. Among the more successful of these is a study by Ramaswamy et al. of molecular signatures of metastasis in primary solid tumors aiming to elucidate the molecular nature of metastasis [7]. The authors analyzed the gene-expression profiles of 12 metastatic adenocarcinoma nodules of diverse origin (lung, breast, prostate, colorectal, uterus, ovary) and compared them with the expression profiles of 64 primary adenocarcinomas representing the same spectrum of tumor types obtained from different individuals. They identified 128 genes differentially expressed between primary and metastatic adenocarcinomas. A similar pattern was found in some primary tumors, which suggests that a gene expression program for metastatic transformation is present in some primary solid tumors. Further refining produced a subset of 17 unique genes that the authors presented as the most significant contributors to the difference between primary and metastatic tumors and thus the most likely diagnostic markers for the metastatic potential.

In this work, we present an alternative analysis of gene expression data based on a holistic approach integrating fragmented biological evidence and strengthening the unreliable conclusions by bringing more data rather than cutting straight to a few most consistent observations. We start with the analysis of the same meta-set of metastatic and primary tumors utilized by Ramaswamy et al., but supplement the analysis by algorithms, methodology and data not available to the original authors.

### Data

**The Ramaswamy *et al. *meta-set **combines genes represented by different probes across multiple distinct microarray platforms (Affymetrix U95A, Hu6800 and Hu35K oligonucleotide microarrays as well as Rosetta inkjet microarrays) traced through by mapping to UniGene build #147. The data have been scaled to account for different microarray intensities in a given set. Each column (sample) has been multiplied in the data set by 1/slope of a least-squares linear fit of the sample versus a reference (the first sample in the data set) using only genes that had 'Present' calls in both the sample being re-scaled and the reference. A typical sample (that is, one with the closest number of 'Present' calls to the average over all samples in the data set) was chosen as reference.

The authors performed thresholding using a ceiling of 16,000 units and a floor of 20 units then subjected gene-expression values to a variation filter that excluded genes with minimal variation across the samples being analyzed by testing for a fold-change and absolute variation over samples, comparing max/min and max - min with predefined values and excluding genes not obeying both conditions. The resulting data are available at .

### Colorectal cancer data sets

The GDS756 dataset provided insight the progression of cancer from primary tumor growth to metastasis by comparison of gene expression in SW480, a primary tumor colon cancer cell line, to that in SW620, an isogenic metastatic colon cancer cell line. Both cell lines were derived from one individual. The GDS1780 set reflects comparison of polysomal RNA from isogenic cell lines established from a colorectal cancer (CRC) patient [6]. The cell lines constitute a cellular model of CRC transition from invasive carcinoma to metastasis. The RNA samples were submitted to microarray analysis using the HG-U133A chip from Affymetrix, (Santa Clara, CA). Three biological replicates were carried out for each cell line and six hybridized arrays obtained. Raw data were analyzed using two microarray analysis software packages, dChip (13) and R-Robust Microarray Analysis (R-RMA) (14). We have downloaded and used these data sets from GEO (GDS756 and GDS1780). Each data set contains 22283 features (probesets) and 6 columns (samples) representing two contrast classes, each with three replicate experiments.

### Breast cancer data

The data set we used in this study was downloaded from the GEO database (GDS2617); it contains 22283 probe sets. Tumorigenic and non-tumorigenic breast cancer cells were compared. Tumorigenic breast cancer cells were considered those expressing cell-surface proteins CD44 and CD24. Tumorigenic breast cancer cells isolated from 6 individuals were compared with normal breast epithelium derived from 3 individuals. In terms adopted by the authors of the original paper [4], tumorigenic cancer samples are those with invasive potential, resulting in metastatic progression.

## Methods

### Overview of the analysis pipeline

The general overview of the analysis pipeline is given in Supplemental Figure 1 (Additional File 1). Our pipeline includes most of the standard analysis steps, but has a few important differences. We extend the analysis to maximize the advantage of pathway analysis. The genes important for understanding the biological processes involved in metastatic transformation are selected not solely by the difference in signal emitted by microarray probes. Instead, we concentrate on the "group behavior" of genes, their ability to interact and pre-existing annotation placing the genes into the same biological pathway, linking to the same cellular function. Thus, the inference is done with very liberal selection criteria and not adjusted for multiple testing. We select a large list of potentially differential genes which may contain a large number of false-positives. We then select biological pathways, molecular function and GO terms which are over-represented in the initial intensity-based list. The benefits of the use of pathway and ontological analyses of microarray data have been presented previously [15,16]. More recent GSEA [17] and SAFE [18] methods can be very effective in highlighting the joint effect of a group of genes which may not be significantly differential if considered one by one. However, these methods require additional assumptions that may not be correct in every study. The significance of biological pathways is estimated through a variation of Fisher's exact test as implemented in Metacore or IPA and adjusted for multiple testing using Benjamini-Hochberg FDR analysis (which is a build-in function of GeneGo Metacore software). Single genes that do not map into any statistically significant pathway (i.e. missing all regulators, downstream targets, ligands and other components necessary for a functional molecular mechanism) may be still considered significant if reproducible and independently validated in additional experiments. However, in our analysis pipeline we leave such genes out regardless of their individual difference between primary and metastatic samples in a particular experiment. Our approach is based on collective effects of the groups of genes interlinked by functional relationships, which is inapplicable to some genes lacking information on function, regulation and interaction with other genes.

**Figure 1:**
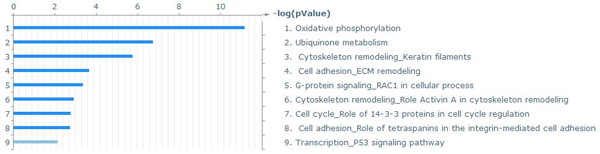
Biological pathways significantly overrepresented in the shortlist of genes differentially expressed between primary solid and metastatic tumors (Ramaswamy et al. data set).

#### Normalization

The data were normalized using a quantile algorithm similar to one described by Bolstad *et al. *[19]. We applied our own C++ software for normalization, available from A. Ptitsyn upon request. Box-plots for pre-normalized and normalized expression value distributions are shown in Supplemental Figure 2 (Additional file 1).

**Figure 2:**
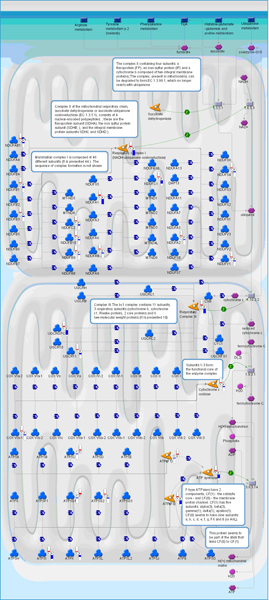
**Genes differentially expressed between primary and metastatic cancers in the oxidative phosphorylation pathway**. Relative change and direction of change in transcript abundance of differentially expressed are marked with color flags. Red color designates higher and blue color designates lower transcript abundance compared to average between primary tumor (1) and metastatic samples (2). The legend for GeneGo pathway maps is given in Supplemental Figure 6 (Additional File 1).

#### Preliminary selection of differentially expressed genes

A set of differentially expressed genes was selected using University of Pittsburgh Gene Expression Data Analysis suite (GEDA, ). For selection, we applied the standard J5 metric with threshold 4 and optional 4 iteration of Jackknife procedure to reduce the number of false-positive differential genes [20]. Both J5 metric and threshold parameter are standard pre-set values recommended by the developers. We did not attempt to estimate the confidence level of individual genes and used J5 not as a statistical test, but as a selection procedure providing a shortlist of genes deviating from the expected average value and enriched with differential genes. The MA plot showing selected differential genes is presented in Supplemental Figure 3 (Additional File 1). Notably, the plot shows a balanced representation of moderately and highly expressed genes, i.e. the categories most appropriate for selection of diagnostic biomarkers. Application of selection procedures biased away from highly expressed genes may reveal truly differential genes, but fewer suitable biomarker candidates. We then applied DAVID web-based tools to perform functional annotation of all potentially differential genes selected by GEDA. The complete annotated lists for analyzed data sets are given in the Supplemental Materials (Supplemental Tables 2, 3, 4 and 5 found in Additional File 1).

**Figure 3:**
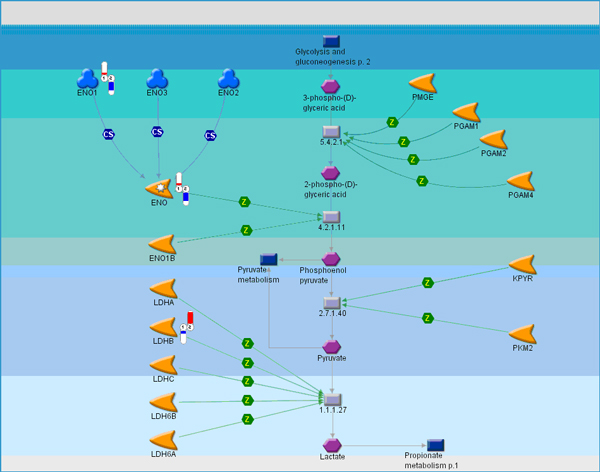
**Glycolysis pathway**. In spite of the fragmentary nature of the composed meta-set, the Warburg effect is still reflected in the pathway map through increased abundance of lactate dehydrogenase (LDHB). Relative change and direction of change in transcript abundance of differentially expressed are marked with color flags. Red color designates higher and blue color designates lower transcript abundance compared to average between primary tumor (1) and metastatic samples (2). The legend for GeneGo pathway maps is given in Supplemental Figure 6 (Additional File 1).

#### Functional annotation and pathway analysis

Analysis of biological pathways was performed using MetaCore software (GeneGo Inc.), Ingenuity Pathways Analysis (Ingenuity Systems Inc.) and free DAVID tools [21].

## Results and discussion

Analysis of the Ramaswamy *et al*. meta-set identified 741 genes differentially expressed between 64 primary solid tumor samples and 12 metastatic tumor samples. The complete list of these genes with functional annotation is given in Supplementary Table 1 (Additional File 1). As expected (see explanation in Methods section), this list is much larger than the original 128 genes identified by Ramaswamy et al. It is likely that there are some false positive differential genes mixed in, however the exact number is not relevant to the analysis. Instead, we focused on the biological function of the genes on the selected shortlist. This function can be estimated through the analysis of the biological pathways, canonic interaction maps and gene ontology categories found within the shortlist. Analysis of statistically overrepresented pathways in the shortlist of differential genes revealed 19 canonic pathway maps (by GeneGo Metacore version) with confidence level *p *= 0.05 (adjusted for FDR). The chart of overrepresented metabolic maps is given on Figure 1. Analysis of the same shortlist of differential genes with the DAVID Functional Classification tool [21] also reveals 6 clusters of gene functions with 3 to 6 members-functional categories (GO terms, PIR keywords, etc.) significantly overrepresented with *p *< 0.05 after FDR (Benjamini-Hochberg) adjustment. These results, presented in Supplemental Table 1 (Additional File 1), are based on algorithms and knowledge bases different from those of GeneGo Metacore. However, scrutinizing the contents of these results allows reconstruction of the underlying biological processes, which are common, robust and reproducible in experiments.

The most remarkable among the pathways differentially represented between primary and metastatic tumors are extracellular matrix/cell adhesion/cytoskeleton remodeling and oxidative phosphorylation. The most common pathways break into three classes: a) related to remodeling of internal cellular structure; b) related to alterations in cellular metabolism; and c) alternations in cell surface, antigen presentation and cell adhesion. Pathways related to cell cycle regulation are also found among differential genes.

Detailed analysis of each overrepresented pathway would be far beyond the scope of this study. However, it is appropriate to comment on key processes reflected by the metabolic maps.

One of the most strongly and consistently altered pathways in all evaluated datasets involves glucose utilization, specifically down-regulation of major components of oxidative phosphorylation (Figure 2) and up-regulation of genes in the glycolytic pathway (Figure 3). Down-regulated genes included mitochontrial ATPase pathway members, cytochrome oxidase, and NADH dehydrogenases. The phenomenon of prefential use of glycolysis for ATP generation in tumors was first observed by Otto Warburg in the first half of the 20^th ^century (as early as 1925) [22-24]. However, more recent studies have demonstrated that exaggeration of the Warburg phenomenon through inhibition of mitochondrial function may promote metastasis via enhancement of tumor cell invasion and reduced sensitivity to apoptosis [25-27]. Furthermore, other groups have recently demonstrated reduction in oxidative phosphorylation-related genes in metastases versus primary tumors using genomic methods [28,29] and correlation of reduction in ATP synthase function with outcome in patients with lung and colorectal cancer [30,31].

In addition to providing a potential novel marker for metastatic potential, the broad conservation of alterations in bioenergetic pathways in metastatic tumors across tumor types and datasets suggests that interference with glycolytic pathways might be a viable therapeutic strategy for the prevention of metastasis. Glycolytic pathway analogs such as 2-deoxyglucose and 3-bromopyruvate are showing promise as therapeutic agents targeting hypoxic primary tumor cells [32], but have been poorly evaluated as antimetastatic drugs. However, a recent study demonstrated inhibition of pancreatic cancer metastasis in mice treated with 3-bromopyruvate when combined with a heat shock protein 90 inhibitor [33]. Furthermore, epigenetic therapies such as histone deacetylase and DNA methyltransferase inhibitors have been shown to reactivate expression of oxidative phosphorylation genes [34], conceivably reducing metastatic potential and suggesting that some alterations in this pathway may be epigenetically regulated.

Another critical pathway in our analysis that was differentially expressed robustly in primary versus metastatic tumors involves the extracellular matrix, cell adhesion, adhesion-mediated signal transduction and cytoskeletal organization, all of which are cooperatively important in the metastatic cascade.

**Figure 4:**
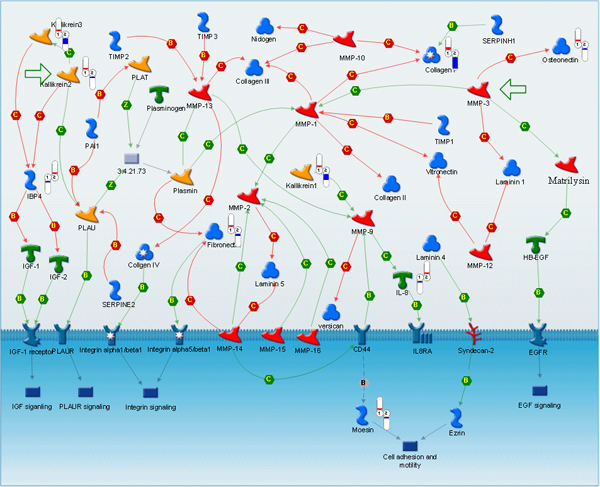
**Alterations in extraceullular matrix and secreted proteins associated with metastatic cancer**. Relative change and direction of change in transcript abundance of differentially expressed are marked with color flags. Red color designates higher and blue color designates lower transcript abundance compared to average between primary tumor (1) and metastatic samples (2). The legend for GeneGo pathway maps is given in Supplemental Figure 6 (Additional File 1).

Alterations in extracellular matrix proteins included reductions in collagen, fibronectin, and a shift in keratin isoform expression (Figure 4). These reductions in cell matrix proteins could theoretically facilitate cell motility and enhance extravasation. The cell adhesion molecules CD63 and CD151 were upregulated in metastatic tumors as well. Experimental and clinical literature demonstrates a role for CD151 in metastasis [35,36]

**Figure 5:**
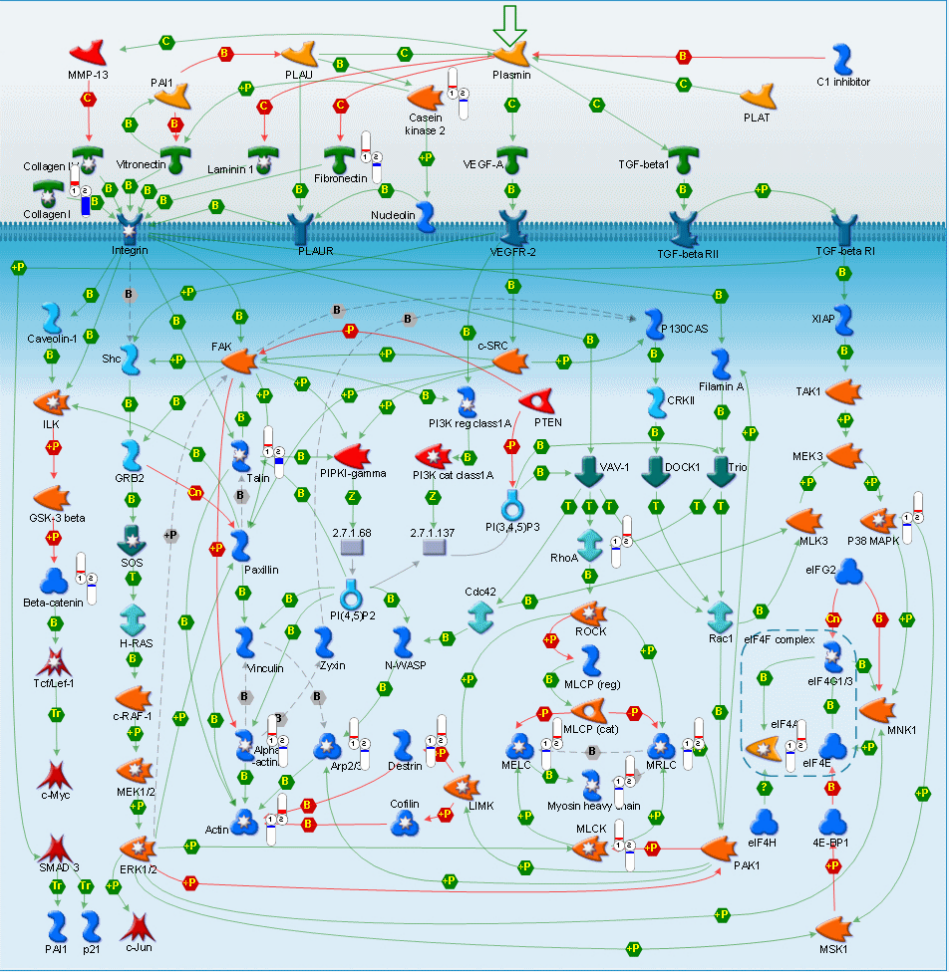
**Alterations in adhesion-mediated signaling and cytoskeleton remodeling in metastatic cancer**. Relative change and direction of change in transcript abundance of differentially expressed are marked with color flags. Red color designates higher and blue color designates lower transcript abundance compared to average between primary tumor (1) and metastatic samples (2). The legend for GeneGo pathway maps is given in Supplemental Figure 6 (Additional File 1).

Differential expression of some key proteins responsible for adhesion-mediated cell signaling (RhoA, talin, moesin, ezrin, SPARC) was also observed (Figure 5). Encouragingly, up-regulation of some well-characterized metastasis-associated genes such as RhoA and ezrin was observed. RhoA plays a key role in regulating the actin cytoskeleton and controlling cell motility, cell-cell interactions and intracellular trafficking [37]. Upregulation of RhoA has been associated with metastasis and/or negative outcome in carcinomas of the liver, kidney, esophagus, and urinary tract [38-40]. Upregulation of ezrin has been implicated in metastasis of diverse tumor types, such as osteosarcoma, soft-tissue sarcomas, pancreatic carcinoma, and head and neck carcinoma among others [41-46].

Significant upregulation of important cytoskeletal components such as actin, tubulin and vimentin was also observed. These proteins play a key role in cell motility, invasion, cell division and intracellular transport, and differential expression of these members has been implicated in human tumor progression as well [47,48]. Increased vimentin is a well-defined phenotypic indicator of epithelial-mesenchymal transition, which has a known association with carcinoma aggressiveness [48].

Several components of the extracellular matrix – cell adhesion – adhesion-mediated signaling – cytoskeleton pathway have the potential for "druggability". For example, small molecule inhibitors of RhoA are in development [49,50], and rapamycin and its analogs have been shown to inhibit the ezrin-associated metastatic phenotype through inhibition of downstream AKT-mTOR signaling [51].

**Figure 6:**
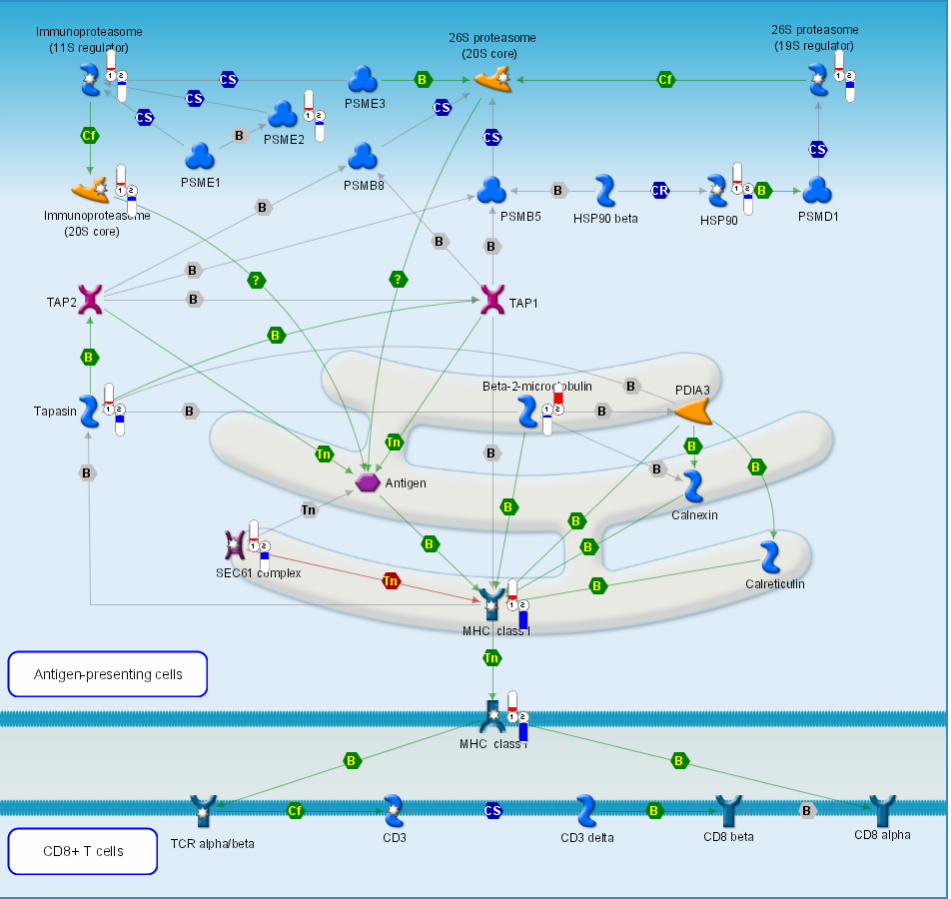
**Alterations in the antigen presentation pathway observed in metastatic tumors**. Relative change and direction of change in transcript abundance of differentially expressed are marked with color flags. Red color designates higher and blue color designates lower transcript abundance compared to average between primary tumor (1) and metastatic samples (2). The legend for GeneGo pathway maps is given in Supplemental Figure 6 (Additional File 1).

The antigen presentation pathway in Figure 6 also reflects, in part, cytoskeleton remodeling: metastatic samples show increased expression of beta-2-microtubulin in the endoplasmic reticulum. All other elements of the antigen presentation pathway found in the differential genes shortlist are down-regulated. Remarkably, the most down-stream elements of the pathway, the final effectors, are the most down-regulated. Immune avoidance is thought to be another key component in successful metastasis; tumor cells must be able to survive in the circulation and avoid immune destruction upon arrest in the end-organ. Furthermore, evidence exists for epigenetic suppression of antigen presentation in tumor cells, and potential reactivation of expression through drugs blocking histone deacetylase and/or DNA methyltransferase, leading to enhanced tumor cell immunogenicity [52-54].

How reproducible are the results of computational analysis of an artificial meta-set of primary and metastatic tumors? We cannot possibly repeat the sample collection, RNA extraction and hybridizations. However, since the time Ramaswamy et al. have published their results there have been quite a few publications reporting microarray analysis of primary vs. metastatic tumors, and the data are available from the public databases such as GEO . We have extracted and analyzed a few of these new data sets [4,6] using the same analysis pipeline. The final results of these analyses are lists of statistically significant pathways, molecular functions and GO terms within the shortlist of potentially differential genes. These lists show remarkable agreement in all studies. Comparison of the pathways represented in these lists reveals none unique to any of the 3 data sets, only common and similar (Supplemental Figure 4 in Additional File 1). Overall, the Ramaswamy et al. meta-set produces a shorter list of potentially differential genes and further analysis yields fewer significant pathways. This result is not surprising taking into account that many small differences reproducibly observed in each single-tissue experiment have been leveled in composing the meta-set. However, the essential features reflecting the metabolic changes between primary and metastatic tumors are apparent in every analyzed data set. The oxidative phosphorylation pathways with most components down-regulated, cytoskeleton remodeling and cell adhesion-related pathways are always found among the longer lists of significant pathways in the specific colon and breast cancer datasets. Remarkably, the suppressed oxidative phosphorylation pathway is always near the top of the most statistically significant pathways.

Taken together, there are dramatic changes in gene expression between primary and metastatic tumors; some are quantitative whereas others reflect a new pattern of expression. But how consistently are those changes revealed by a loose non-parametric J5 procedure? This selection procedure gives no estimation of confidence level for the individual genes. In turn, estimation of significance for the biological pathways is very approximate at best: it does not fully account for interdependencies in gene expression. Pathway maps include genes arbitrarily and the database of gene interactions is filled manually by multiple experts scanning the peer-reviewed literature, i.e. prone to errors and contradictions. These databases and associated tools for pathway analysis have improved significantly in recent years, but quantitative estimation of pathway significance still needs additional validation. In order to select only the most reliably over-represented pathways, we performed a bootstrap analysis randomly re-sampling 50% of the short-listed genes. Comparative analysis of over-represented pathways in the randomly re-sampled and original shortlists is given in Supplemental Table 6 and Supplemental Figure 5 (Additional File 1). The main pathways are remarkably robust. The genes (putative biomarkers) diagram is dominated by "similar" pathways, i.e. belonging to the same pathway map or involved in the same cellular function. There are also some "common" genes (i.e. genes representing the same pathway, which is still statistically significant in the randomly selected half-list) and no "unique" genes (i.e. representing unique, but statistically significant pathways). This observation leads to important conclusions: a) microarray experiments may yield extensive variation in specific differentially expressed genes, but are robust and reproducible in elucidating differentially expressed pathways; b) random re-sampling of the large list of differentially expressed genes provides no proof of true difference for any single gene, but the list in general has few (if any) false-positive genes. The latter statement is controversial since the common goal of the inference in microarray analysis is to reduce the dimensionality of the feature space and select a small number of truly differential genes. After selection of a shortlist using a *t*-test or one of its variants, the number of differentially expressed genes is further reduced by application of a False Discovery Rate procedure (typically Benjamini-Hochberg) [55,56]. Some authors even claim that microarrays are not optimal for pathway analysis because of poor reproducibility of the resulting pathways [57]. Our study suggests the opposite. The previously discussed problem of pathway reproducibility is caused by the misconceived methodology, more specifically in the strategy of microarray data analysis. Apparently, applying stricter criteria for selection of differentially expressed genes results in a very small number of candidates that are further reduced by FDR adjustment. The few remaining candidate genes have a much better chance of being successfully reproduced in another microarray experiment and validated by other techniques such as real-time RT-PCR or immunohistochemistry. On the other hand, a shorter list of candidate genes undermines the basis for the pathway analysis, rendering overrepresentation statistics powerless. This may explain the poor reproducibility of pathway analysis in some studies [57]. Such a stringent approach to biomarker selection relies entirely on the signal intensity and associated statistics. This approach can be very effective in cases of lethal mutations, congenital disorders and other diseases caused by a single or few factors. However, in complex multifactorial diseases, the most highly expressed genes and most reproducible differences in gene expression often turn out to be non-specific final effectors, downstream of important switches and regulators in biological pathways. Cancer in general and metastasis in particular are the examples of such multifactorial diseases. Application of a systems biology approach, considering not just the effect of single mutated/healthy genes, but entire networks of interlinked and constantly interacting genes is required not only for understanding the mechanism of disease, but also for the selection of diagnostic and prognostic markers, as well as potential therapeutic targets. As we have demonstrated, the pathways are sufficiently reproducible and robust to serve this purpose. The prevailing methodology in microarray analysis has an internal contradiction: it calls for a strict selection of candidate genes that can be independently verified one by one, but systems biology calls for analysis of large numbers of genes. Furthermore, the number of replicates affordable for a typical microarray study is usually insufficient for reliable reproduction of expression in low-expressed genes. However, important biological functions specific to disease are often performed by low-expressed genes. Pathway analysis has the power to identify such signal transducers and key transcription factors only if a large enough number of candidate genes are input. To resolve this contradiction, we propose an extension of the current prevailing methodology.

First, the analysis pipeline has to be extended to incorporate functional annotation and pathway analysis. Second, selection of the candidate genes cannot be performed based solely on the intensity of signal and its change in the experiment. Instead, we propose to consider this step a pre-selection and relax the criteria for "differential" genes. Third, FDR correction should not be applied to a pre-selected "long list" of candidate genes. Combined with a relaxed selection threshold, this will inevitably create an influx of false-positive genes, which can be addressed subsequently. Fourth, the "long list" is analyzed in order to identify statistically overrepresented biological pathways, GO terms, molecular functions (as implemented in DAVID, IPA and MetaCore software) and gene set enrichment (for example, using GSEA or SAFE methods [17,18]). It is at this stage of analysis that multiple testing adjustments (Bonferroni, or better FDR) should be applied. Most available software, both free (DAVID tools [21]) and commercial (such as IPA and Metacore) have at least one method of false-positive control implemented. However, we still recommend additional techniques, such as the bootstrapping experiment described above, for computational validation of significant pathways. Finally, the discovered statistically significant pathways, gene sets and molecular functions should be used to reverse-engineer the molecular mechanism of disease and select one or more potential biomarkers and drug targets. In our approach, it is important to combine numeric analysis with biological reasoning and deduction.

The proposed analysis strategy is not yet implemented in a single analysis tool, although all the components have been developed and some of the software packages (such as ArrayTrack [58]) offer partial integration; pathway analysis packages, although independent, can be easily invoked from within the microarray analysis software. In the future, we would like to unite all the tools used for systems biology analysis of biomarkers in a single automated software pipeline.

Systems biology approaches to analysis of existing public data reveal a large number of new features overlooked in the original analyses. Meta-analysis and cross-examination of a few data sets allows identification of prospective markers and drug targets. The present day databases available for systems biology empower the researchers beyond the dreams of only a few years ago. Now for each identified significant pathway, we may also correlate expression data with known conserved transcription factor binding sites, and employ siRNA-mediated gene knockdown and known pharmacologic inhibitors (pharmacoprobes) to interrogate the phenotypic effects of interference with identified pathways. The systems approach described here allows identification of a number of key pathways that may serve as therapeutic targets for controlling the metastatic transition of primary solid tumors.

## Competing interests

The authors declare that they have no competing interests.

## Authors' contributions

AAP has collected the data, developed the algorithms, the software and performed data analysis. AAP, MMW and DHT interpreted the results and wrote the paper.

## Supplementary Material

Additional file 1 Supplementary figures 1–6 and Supplementary tables 1–6. Click here for file

## References

[B1] Onn A, Fidler IJ (2002). Metastatic potential of human neoplasms. In Vivo.

[B2] Fidler IJ (2003). The pathogenesis of cancer metastasis: the 'seed and soil' hypothesis revisited. Nat Rev Cancer.

[B3] Fidler IJ, Kripke ML (1977). Metastasis results from preexisting variant cells within a malignant tumor. Science.

[B4] Liu R, Wang X, Chen GY, Dalerba P, Gurney A, Hoey T, Sherlock G, Lewicki J, Shedden K, Clarke MF (2007). The prognostic role of a gene signature from tumorigenic breast-cancer cells. N Engl J Med.

[B5] Parker B, Sukumar S (2003). Distant metastasis in breast cancer: molecular mechanisms and therapeutic targets. Cancer Biol Ther.

[B6] Provenzani A, Fronza R, Loreni F, Pascale A, Amadio M, Quattrone A (2006). Global alterations in mRNA polysomal recruitment in a cell model of colorectal cancer progression to metastasis. Carcinogenesis.

[B7] Ramaswamy S, Ross KN, Lander ES, Golub TR (2003). A molecular signature of metastasis in primary solid tumors. Nat Genet.

[B8] van 't Veer LJ, Dai H, Vijver MJ van de, He YD, Hart AA, Mao M, Peterse HL, Kooy K van der, Marton MJ, Witteveen AT (2002). Gene expression profiling predicts clinical outcome of breast cancer. Nature.

[B9] Fidler IJ (1995). Modulation of the organ microenvironment for treatment of cancer metastasis. J Natl Cancer Inst.

[B10] Hunter K (2006). Host genetics influence tumour metastasis. Nat Rev Cancer.

[B11] Crawford NP, Hunter KW (2006). New perspectives on hereditary influences in metastatic progression. Trends Genet.

[B12] LaTulippe E, Satagopan J, Smith A, Scher H, Scardino P, Reuter V, Gerald WL (2002). Comprehensive gene expression analysis of prostate cancer reveals distinct transcriptional programs associated with metastatic disease. Cancer Res.

[B13] Scher HI, Heller G (2000). Clinical states in prostate cancer: toward a dynamic model of disease progression. Urology.

[B14] Fidler IJ, DeVita VT, Hellman, S, Rosenberg SA (1997). Molecular biology of cancer: invasion and metastasis. Cancer Principles and Practice of Oncology.

[B15] Draghici S, Khatri P, Martins RP, Ostermeier GC, Krawetz SA (2003). Global functional profiling of gene expression. Genomics.

[B16] Manoli T, Gretz N, Grone HJ, Kenzelmann M, Eils R, Brors B (2006). Group testing for pathway analysis improves comparability of different microarray datasets. Bioinformatics.

[B17] Subramanian A, Tamayo P, Mootha VK, Mukherjee S, Ebert BL, Gillette MA, Paulovich A, Pomeroy SL, Golub TR, Lander ES (2005). Gene set enrichment analysis: a knowledge-based approach for interpreting genome-wide expression profiles. Proc Natl Acad Sci USA.

[B18] Barry WT, Nobel AB, Wright FA (2005). Significance analysis of functional categories in gene expression studies: a structured permutation approach. Bioinformatics.

[B19] Bolstad BM, Irizarry RA, Astrand M, Speed TP (2003). A comparison of normalization methods for high density oligonucleotide array data based on variance and bias. Bioinformatics.

[B20] Patel S, Lyons-Weiler J (2004). caGEDA: a web application for the integrated analysis of global gene expression patterns in cancer. Appl Bioinformatics.

[B21] Huang da W, Sherman BT, Tan Q, Collins JR, Alvord WG, Roayaei J, Stephens R, Baseler MW, Lane HC, Lempicki RA (2007). The DAVID Gene Functional Classification Tool: a novel biological module-centric algorithm to functionally analyze large gene lists. Genome Biol.

[B22] Warburg O (1925). Uber den Stoffwechsel der Carcinomzelle. Klin Wochenschr.

[B23] Warburg O (1956). On the origin of cancer cells. Science.

[B24] Warburg O (1956). On respiratory impairment in cancer cells. Science.

[B25] Amuthan G, Biswas G, Ananadatheerthavarada HK, Vijayasarathy C, Shephard HM, Avadhani NG (2002). Mitochondrial stress-induced calcium signaling, phenotypic changes and invasive behavior in human lung carcinoma A549 cells. Oncogene.

[B26] Harris MH, Vander Heiden MG, Kron SJ, Thompson CB (2000). Role of oxidative phosphorylation in Bax toxicity. Mol Cell Biol.

[B27] Dey R, Moraes CT (2000). Lack of oxidative phosphorylation and low mitochondrial membrane potential decrease susceptibility to apoptosis and do not modulate the protective effect of Bcl-x(L) in osteosarcoma cells. J Biol Chem.

[B28] Gmeiner WH, Hellmann GM, Shen P (2008). Tissue-dependent and -independent gene expression changes in metastatic colon cancer. Oncol Rep.

[B29] Lin HM, Chatterjee A, Lin YH, Anjomshoaa A, Fukuzawa R, McCall JL, Reeve AE (2007). Genome wide expression profiling identifies genes associated with colorectal liver metastasis. Oncol Rep.

[B30] Cuezva JM, Chen G, Alonso AM, Isidoro A, Misek DE, Hanash SM, Beer DG (2004). The bioenergetic signature of lung adenocarcinomas is a molecular marker of cancer diagnosis and prognosis. Carcinogenesis.

[B31] Cuezva JM, Krajewska M, de Heredia ML, Krajewski S, Santamaria G, Kim H, Zapata JM, Marusawa H, Chamorro M, Reed JC (2002). The bioenergetic signature of cancer: a marker of tumor progression. Cancer Res.

[B32] Airley RE, Mobasheri A (2007). Hypoxic regulation of glucose transport, anaerobic metabolism and angiogenesis in cancer: novel pathways and targets for anticancer therapeutics. Chemotherapy.

[B33] Cao X, Jia G, Zhang T, Yang M, Wang B, Wassenaar PA, Cheng H, Knopp MV, Sun D (2008). Non-invasive MRI tumor imaging and synergistic anticancer effect of HSP90 inhibitor and glycolysis inhibitor in RIP1-Tag2 transgenic pancreatic tumor model. Cancer Chemother Pharmacol.

[B34] Duenas-Gonzalez A, Candelaria M, Perez-Plascencia C, Perez-Cardenas E, de la Cruz-Hernandez E, Herrera LA (2008). Valproic acid as epigenetic cancer drug: Preclinical, clinical and transcriptional effects on solid tumors. Cancer Treat Rev.

[B35] Hong IK, Jin YJ, Byun HJ, Jeoung DI, Kim YM, Lee H (2006). Homophilic interactions of Tetraspanin CD151 up-regulate motility and matrix metalloproteinase-9 expression of human melanoma cells through adhesion-dependent c-Jun activation signaling pathways. J Biol Chem.

[B36] Ang J, Lijovic M, Ashman LK, Kan K, Frauman AG (2004). CD151 protein expression predicts the clinical outcome of low-grade primary prostate cancer better than histologic grading: a new prognostic indicator?. Cancer Epidemiol Biomarkers Prev.

[B37] Merajver SD, Usmani SZ (2005). Multifaceted role of Rho proteins in angiogenesis. J Mammary Gland Biol Neoplasia.

[B38] Wang D, Dou K, Xiang H, Song Z, Zhao Q, Chen Y, Li Y (2007). Involvement of RhoA in progression of human hepatocellular carcinoma. J Gastroenterol Hepatol.

[B39] Takami Y, Higashi M, Kumagai S, Kuo PC, Kawana H, Koda K, Miyazaki M, Harigaya K (2008). The activity of RhoA is correlated with lymph node metastasis in human colorectal cancer. Dig Dis Sci.

[B40] Kamai T, Tsujii T, Arai K, Takagi K, Asami H, Ito Y, Oshima H (2003). Significant association of Rho/ROCK pathway with invasion and metastasis of bladder cancer. Clin Cancer Res.

[B41] Chai LX, Sun KL, Guo LP, Zhang HT, Lu SX (2007). Expression of ezrin and CD44-v6 in human esophageal squamous cell carcinoma and its clinical significance. Zhonghua Zhong Liu Za Zhi.

[B42] Torer N, Kayaselcuk F, Nursal TZ, Yildirim S, Tarim A, Noyan T, Karakayali H (2007). Adhesion molecules as prognostic markers in pancreatic adenocarcinoma. J Surg Oncol.

[B43] Kobel M, Gradhand E, Zeng K, Schmitt WD, Kriese K, Lantzsch T, Wolters M, Dittmer J, Strauss HG, Thomssen C (2006). Ezrin promotes ovarian carcinoma cell invasion and its retained expression predicts poor prognosis in ovarian carcinoma. Int J Gynecol Pathol.

[B44] Yeh TS, Tseng JH, Liu NJ, Chen TC, Jan YY, Chen MF (2005). Significance of cellular distribution of ezrin in pancreatic cystic neoplasms and ductal adenocarcinoma. Arch Surg.

[B45] Weng WH, Ahlen J, Astrom K, Lui WO, Larsson C (2005). Prognostic impact of immunohistochemical expression of ezrin in highly malignant soft tissue sarcomas. Clin Cancer Res.

[B46] Khanna C, Wan X, Bose S, Cassaday R, Olomu O, Mendoza A, Yeung C, Gorlick R, Hewitt SM, Helman LJ (2004). The membrane-cytoskeleton linker ezrin is necessary for osteosarcoma metastasis. Nat Med.

[B47] Kelley LC, Shahab S, Weed SA (2008). Actin cytoskeletal mediators of motility and invasion amplified and overexpressed in head and neck cancer. Clin Exp Metastasis.

[B48] Mandal M, Myers JN, Lippman SM, Johnson FM, Williams MD, Rayala S, Ohshiro K, Rosenthal DI, Weber RS, Gallick GE (2008). Epithelial to mesenchymal transition in head and neck squamous carcinoma: association of Src activation with E-cadherin down-regulation, vimentin expression, and aggressive tumor features. Cancer.

[B49] Xue F, Zhang JJ, Qiu F, Zhang M, Chen XS, Li QG, Han LZ, Xi ZF, Xia Q (7632). Rho signaling inhibitor, Y-2 inhibits invasiveness of metastastic hepatocellular carcinoma in a mouse model. Chin Med J (Engl).

[B50] Evelyn CR, Wade SM, Wang Q, Wu M, Iniguez-Lluhi JA, Merajver SD, Neubig RR (2007). CCG-1423: a small-molecule inhibitor of RhoA transcriptional signaling. Mol Cancer Ther.

[B51] Wan X, Mendoza A, Khanna C, Helman LJ (2005). Rapamycin inhibits ezrin-mediated metastatic behavior in a murine model of osteosarcoma. Cancer Res.

[B52] Khan AN, Magner WJ, Tomasi TB (2007). An epigenetic vaccine model active in the prevention and treatment of melanoma. J Transl Med.

[B53] Khan AN, Gregorie CJ, Tomasi TB (2008). Histone deacetylase inhibitors induce TAP, LMP, Tapasin genes and MHC class I antigen presentation by melanoma cells. Cancer Immunol Immunother.

[B54] Manning J, Indrova M, Lubyova B, Pribylova H, Bieblova J, Hejnar J, Simova J, Jandlova T, Bubenik J, Reinis M (2008). Induction of MHC class I molecule cell surface expression and epigenetic activation of antigen-processing machinery components in a murine model for human papilloma virus 16-associated tumours. Immunology.

[B55] Benjamini YaH Y (1995). Controlling the false discovery rate: a practical and powerful approach to multiple testing. J Roy Stat Soc B.

[B56] Storey JD, Tibshirani R (2003). Statistical methods for identifying differentially expressed genes in DNA microarrays. Methods Mol Biol.

[B57] Adrian Mondry ML, Alessandro Giuliani (2007). DNA expression microarrays may be the wrong tool to identify biological pathways. Nature Preceedings.

[B58] Tong W, Cao X, Harris S, Sun H, Fang H, Fuscoe J, Harris A, Hong H, Xie Q, Perkins R (2003). ArrayTrack – supporting toxicogenomic research at the U.S. Food and Drug Administration National Center for Toxicological Research. Environ Health Perspect.

