# Supplementary material for: Systems biology approach to identification of biomarkers for metastatic progression in cancer
Source: BMC Bioinformatics. 2008 Aug 12;9(Suppl 9):S8. doi: 10.1186/1471-2105-9-S9-S8 (PMC2537559; doi:10.1186/1471-2105-9-S9-S8)
Supplement: Additional file 1 — Supplementary figures 1–6 and Supplementary tables 1–6. [file 1471-2105-9-S9-S8-S1.zip › Supplemental Figure 2.doc]

Supplemental Figure 2. Ramaswami et al. meta-set before and after quantile normalization.

| Primary tumors | Metastatic tumors |
| --- | --- |
| Tumor__Breast_Adeno_09-B_003A, Tumor__Breast_Adeno_09-B_005A, Tumor__Breast_Adeno_92_I_078, Tumor__Breast_Adeno_93_I_192, Tumor__Breast_Adeno_93_I_250, Tumor__Breast_Adeno_94_I_155, Tumor__Breast_Adeno_94_I_159, Tumor__Breast_Adeno_95_I_029, Tumor__Breast_Adeno_9912c068_CC, Tumor__Breast_Adeno_mBRT1_(8697), Tumor__Breast_Adeno_mBRT2_(9078), Tumor__Colorectal_Adeno_0001c026_CC, Tumor__Colorectal_Adeno_0001c038_CC, Tumor__Colorectal_Adeno_0001c040_CC, Tumor__Colorectal_Adeno_95_I_057, Tumor__Colorectal_Adeno_95_I_175, Tumor__Colorectal_Adeno_9912c055_CC, Tumor__Colorectal_Adeno_HCTN_19339, Tumor__Colorectal_Adeno_HCTN_19389, Tumor__Colorectal_Adeno_HCTN_CRT1_(18851_A1B), Tumor__Colorectal_Adeno_mCRT1_(8936), Tumor__Colorectal_Adeno_mCRT2_(9752), Tumor__Lung_Adeno_004_B, Tumor__Lung_Adeno_009_C, Tumor__Lung_Adeno_93_I_146, Tumor__Lung_Adeno_93_I_226, Tumor__Lung_Adeno_94_I_196, Tumor__Lung_Adeno_95_I_117, Tumor__Lung_Adeno_HCTN_LUT1_(18702_A1F), Tumor__Lung_Adeno_HCTN_LUT4_(18870_A1C), Tumor__Lung_Adeno_H_20154, Tumor__Lung_Adeno_H_20387, Tumor__Lung_Adeno_LT14, Tumor__Ovary_Adeno_0001c086, Tumor__Ovary_Adeno_07-B_001B, Tumor__Ovary_Adeno_07-B_014G, Tumor__Ovary_Adeno_93_I_081, Tumor__Ovary_Adeno_HCTN_0002c011N, Tumor__Ovary_Adeno_HCTN_19120, Tumor__Ovary_Adeno_HCTN_19155, Tumor__Ovary_Adeno_H_(400)6346, Tumor__Ovary_Adeno_H_6206, Tumor__Ovary_Adeno_mOVT1_(8691), Tumor__Ovary_Adeno_mOVT2_(I)_(9334), Tumor__Prostate_Adeno_94_I_052, Tumor__Prostate_Adeno_95_I_249, Tumor__Prostate_Adeno_95_I_256, Tumor__Prostate_Adeno_LocalCaP10T, Tumor__Prostate_Adeno_LocalCaP1T, Tumor__Prostate_Adeno_P_0025, Tumor__Prostate_Adeno_P_0030, Tumor__Prostate_Adeno_P_0033, Tumor__Prostate_Adeno_P_0036, Tumor__Prostate_Adeno_P_0062, Tumor__Uterus_2552, Tumor__Uterus_2967, Tumor__Uterus_3226, Tumor__Uterus_3663, Tumor__Uterus_4075, Tumor__Uterus_4203, Tumor__Uterus_4840, Tumor__Uterus_4915, Tumor__Uterus_5116, Tumor__Uterus_92_I_073. | Met__Unknown_CUP_1, Met__Unknown_CUP_16, Met__Unknown_CUP_22,  Met__Unknown_CUP_12,  Met__Unknown_CUP_14,  Met__Unknown_CUP_19,  Met__Metastases_9912c062_Rb, Met__Metastases_HCTN_19274, Met__Metastases_MetCaP109, Met__Metastases_MetCaP125, Met__Metastases_MetCaP128, Met__Metastases_MGH_4934 |
