# Supplementary material for: Systems biology approach to identification of biomarkers for metastatic progression in cancer
Source: BMC Bioinformatics. 2008 Aug 12;9(Suppl 9):S8. doi: 10.1186/1471-2105-9-S9-S8 (PMC2537559; doi:10.1186/1471-2105-9-S9-S8)
Supplement: Additional file 1 — Supplementary figures 1–6 and Supplementary tables 1–6. [file 1471-2105-9-S9-S8-S1.zip › Supplemental Figure 6.doc]

Supplemental Figure 6. The legend for GeneGo Metacore pathway maps.
